# Hospital Nurses' Professional Quality of Life Model: A Cross-Sectional Study Based on the Expanded Job Demands-Resources Model

**DOI:** 10.1155/jonm/7500360

**Published:** 2025-04-07

**Authors:** Younghee Kim, Mi Yu

**Affiliations:** ^1^Department of Nursing, Jeonbuk Science College, Jeongeup, Republic of Korea; ^2^College of Nursing, Sustainable Health Research Institute, Gyeongsang National University, Jinju, Republic of Korea

**Keywords:** career choice, compassion fatigue, nurses, ownership, quality of life

## Abstract

**Aim:** This study aimed to develop a model that explained the factors influencing the professional quality of life (ProQoL) in hospital nurses based on the expanded job demands-resources model (JD-R model).

**Methods:** This cross-sectional study included 296 nurses with > 1 year of experience from three general hospitals in South Korea. Data were collected via self-reported questionnaires between February 13 and 24, 2023. Job stress (JS), supportive organizational environment (SOE), psychological ownership (PO), and career commitment (CC) were exogenous variables. Compassion fatigue (CF) and satisfaction (CS), components of ProQoL, were the endogenous variables. A hypothetical model was assessed through maximum likelihood and bootstrapping via SPSS/AMOS.

**Results:** CF was directly influenced by JS (*β* = 0.44, *p* < 0.001) and CC (*β* = −0.28, *p* < 0.001). CS was influenced by JS (*β* = −0.16, *p*=0.003), PO (*β* = 0.30, *p*=0.012), and CC (*β* = 0.33, *p* < 0.001). The model's explanatory power for CF and CS was 37.0% and 39.0%, respectively. SOE (*β* = −0.15 *p*=0.009) indirectly affected CF through PO and CC. Additionally, PO indirectly affected (*β* = −0.09, *p*=0.008) CF through CC. SOE (*β* = 0.34 *p*=0.014) indirectly affected CS through PO and CC. PO also had an indirect effect (*β* = 0.11, *p*=0.004) on CS through CC. The final model exhibited a good fit.

**Conclusions:** The ProQoL model, based on the expanded JD-R model, is suitable for explaining and predicting the ProQoL among hospital nurses. CC is crucial in mediating the relationships between a SOE, PO, and CF or CS. These findings have implications for developing strategies to enhance nurses' ProQoL.

**Implication for Nursing Management:** This implies the need to reduce JS through workplace improvements, appropriate compensation, and feedback while fostering PO and CC through supportive programs and participatory decision making.

## 1. Introduction

Quality of life is the main focus of the nursing profession, as nurses aim to enhance patients' well-being. However, many nurses may need to fully realize that their work influences their quality of life [1]. While striving to improve patients' quality of life, nurses often face a decline in their own due to fatigue, burnout, and job stress (JS), often without fully recognizing these effects [2]. This challenge is further exacerbated in nursing contexts where moral beliefs and ethical values that prioritize patient care over personal well-being manifest as cultural norms, leading to the sacrifice of one's own health and welfare and potentially increasing nurses' physical and emotional burnout in South Korea [3].

Stamm [4, 5] introduced the concept of professional quality of life (ProQoL), referring to “the quality one feels about their work as a helper.” ProQoL includes three components: compassion satisfaction (CS), compassion fatigue (CF), and burnout, which reflect both positive and negative aspects of caregiving. CF and burnout are used interchangeably, and CF is burnout that is specific to caregivers (nurses, physicians, therapists, etc.) [4, 5]. CS, a positive and altruistic characteristic, is enhanced when caring for and helping others and increasing self-evaluation [5]. The work environment determines it, as well as clients' demands and personal characteristics.

Although helping others can increase CS, those engaged in long-term caregiving may experience JS due to a misalignment between job demands and available resources, leading to CF or burnout [6]. Nurses often experience continuous physical and psychological stress when caring for patients, which decreases CS and increases CF and burnout. Burnout, a component of ProQoL and CF, is a negative side effect of secondary traumatic stress that occurs based on professional job demands and resources. Zhang et al.'s meta-analysis [7] reported that nurses experienced extremely high CF and burnout, which negatively impacted the quality of patient care, patient satisfaction [8], and job satisfaction [9]. Additionally, decreased ProQoL could lead to nurses' turnover [10]. Therefore, assessments of nurses' ProQoL could lead to developing measures to retain them in healthcare institutions [11]. Hence, managing nurses' ProQoL is necessary to retain nurses and maintain the quality of patient care [12]. Therefore, identifying the factors that influence nurses' ProQoL should be prioritized.

A lack of balance between job demands and resources can lead to burnout, psychosomatic complaints, and restrictions in performance [13]. The nursing profession requires physical, psychological, social, and organizational effort. Furthermore, JS caused by various factors, such as heavy workloads, shift work, and time pressure, can lead to burnout [14]. JS also reduces CS [2, 15, 16]. Additionally, a lack of job and personal resources, such as support systems, to overcome or alleviate stress could result in burnout [14].

Conversely, personal resources, such as resilience [14], optimism [17], self-efficacy [16], and organizational support, such as colleagues [16, 18], supervisory [19], and social support [20] and opportunities for career development [17, 20], reduce the adverse effects of job demands, such as JS, and stimulated job motivation. Moreover, resources such as self-care and self-compassion [21] or compassion competence [22] positively impact ProQoL. This prompts individual growth and progress toward the achievement of organizational goals. Moreover, job resources affect career commitment (CC) [23, 24] and are positively and negatively correlated with CS in ProQoL and burnout, respectively [25, 26].

The job demands-resources (JD-R) model, first developed by Demerouti et al. [27], continues to build on its foundational principles, positing that every job involves both job demands and job resources, which influence employee well-being and performance. Job demands, such as high workload or emotional demands, can lead to burnout when they exceed the capacity to recover. By contrast, job resources, such as participation or supervisor support, can boost job engagement or lower disengagement, leading to better performance and well-being. The expanded JD-R model was further developed by Xanthopoulou et al. [17] by incorporating personal resources (e.g., self-efficacy and optimism), exploring how job demands and resources impact exhaustion (burnout) and work engagement, and describes the interplay between job demands, job characteristics that induce stress, job resources that represent a support system, and personal resources. In other words, job resources influence personal resources and work engagement. Additionally, personal resources significantly affect work engagement and partially mediate the relationship between job resources and work engagement, ultimately impacting burnout [17].

Psychological ownership (PO) refers to the feeling that one belongs to or is connected with an organization, influencing how an individual identifies with their work and its values. Previous studies [28, 29] show that PO includes self-efficacy, responsibility, belongingness, and self-identity. These components shape how employees engage with their work environment and perceive their role within it while positively affecting job satisfaction, organizational commitment, work engagement, and reduced burnout [30, 31]. Work engagement, or “involvement, commitment, passion, and enthusiasm,” refers to the desire to remain with an organization [32] or a positive attitude toward one's work [33]. Such work engagement positively and negatively correlated with CS in ProQoL and burnout, respectively [17]. CC, the motivation to work in one's chosen profession, exceeds mere attachment to the profession and refers to the will, loyalty, and dedication to continue one's career path aligned with developing and advancing one's career [34]. Additionally, it was seen as sharing context with work engagement.

In South Korea, research involving the JD-R model [14, 16, 20] was limited to nurses in intensive care units, nursing homes, and advanced general hospitals. Furthermore, research focused on only a few related variables, such as burnout. Identifying domestic studies that validated ProQoL based on this model was difficult. However, it is necessary to explore the factors that allow nurses to maintain their careers long-term and improve and sustain their quality of life. Comprehensive research is required to propose individual, organizational, and institutional enhancement strategies, especially regarding job demands and resources.

Accordingly, this study aimed to provide foundational data to support the retention of experienced nurses and to develop effective human resource management strategies by analyzing factors related to ProQoL for nurses in previous research. Moreover, this study analyzed the impact of job demands, including JS; job resources, like a supportive organizational environment (SOE); and personal resources, such as PO, on CC and ProQoL among hospital nurses based on the expanded JD-R model [17]. In brief, this study aimed to ascertain the levels of JS, SOE, PO, CC, and ProQoL among nurses and clarify their structural relationship as factors that influenced ProQoL. This study will establish a model for the ProQoL of experienced hospital nurses, contributing to the further expansion of the theoretically grounded JD-R model.

## 2. Conceptual Framework and Hypothetical Model of the Study

The conceptual framework was based on the expanded JD-R model [17, 26], which posits that the interaction between job demands and job resources influences employee outcomes. In this framework, job demands, such as high workload or JS, are factors that increase ProQoL's CF [14, 15] and decrease CS [2, 15]. Conversely, job resources, such as social support, participation, and organizational climate, contribute positively by increasing ProQoL's CS [35], reducing CF [35], and enhancing CC [24]. These resources also indirectly influence personal resources, such as self-efficacy or resilience [36], which in turn are associated with improved CC [37], increased CS, and reduced CF [31]. Finally, CC, a mediator in this framework, enhances CS [25] and mitigates CF [24, 38].

In this study, job demands were conceptualized as JS, and job resources were defined as SOEs that included organizational support, supervisor support, and peer support. Personal resources were defined as PO, encompassing self-efficacy, responsibility, belonging, and self-identity.

**Figure 1:**
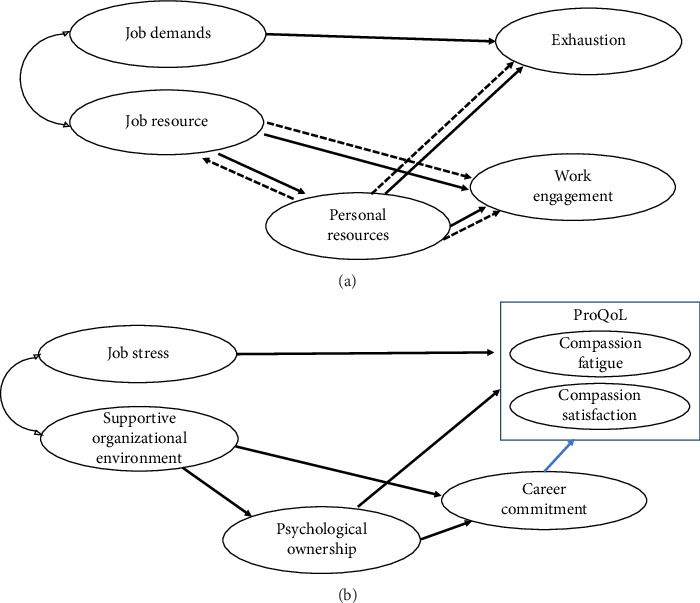
(a) Expanded job demands-resources model by Xanthopoulou et al. [17]. (b) Conceptual framework for nurse's professional quality of life based on expanded job demands-resources model.

Therefore, paths were established in which job directly affected ProQoL (CS and CF): a SOE impacted PO and CC, PO affected CC and ProQoL, and CC directly affected ProQoL. Therefore, the hypothesized model comprised two exogenous (JS and SOE) and four endogenous (PO, CC, and ProQoL; CF and CS) variables (Figure 1).

## 3. Methods

### 3.1. Study Design

This cross-sectional descriptive study established pathways through which JS, SOE, PO, and CC affected nurses' ProQoL and verified the model's fit and hypotheses. This study was conducted in accordance with the STROBE (Strengthening the Reporting of Observational Studies in Epidemiology) checklist for the reporting of observational studies.

### 3.2. Participants

**Figure 2:**
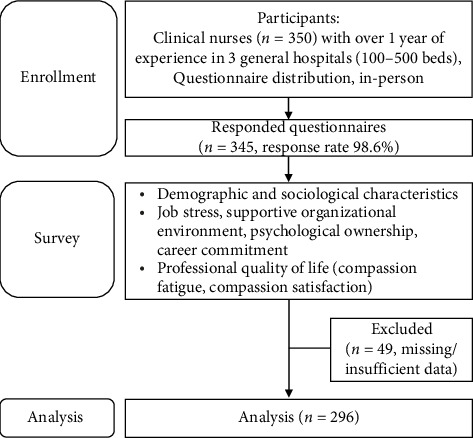
Study sampling.

Participants were clinical nurses who had worked for > 1 year in three general hospitals located in ⁣^∗^City, ⁣^∗^Province, and ⁣^∗^Province, each with > 100 and < 500 beds. Inclusion criteria were those who worked in general wards, intensive care units, operating rooms, delivery rooms, and emergency rooms, understood the study purpose and intent, and provided written consent. Exclusion criteria were nurses with < 1 year of employment experience, outpatient departments, and nurse managers. The sample size was considered appropriate based on the structural equation modeling guidelines, which suggested a sample size of approximately 150–400. Considering the sample size ratio to free parameters, a path coefficient ratio of approximately 1:20 [39], 350 questionnaires were distributed, and 345 (response rate 98.6%) responses were obtained. After 49 responses were excluded owing to missing or insufficient responses, 296 were analyzed and deemed an adequate sample size (Figure 2).

### 3.3. Measurements

#### 3.3.1. Demographic and Sociological Characteristics

Items included gender, age, educational level, marital status, number of hospital beds, position, work pattern, work unit, total clinical experience, current unit experience, satisfaction with the hospital's welfare, and job satisfaction.

#### 3.3.2. JS

JS was measured via the Korean Occupational Stress Scale (KOSS-26), revised by Baik and Choi-Kwon [40] for shift-working nurses. The KOSS-26 comprised 26 items across eight domains: physical environment (two items), job demands (four items), lack of job autonomy (four items), interpersonal conflict (three items), job insecurity (two items), organizational system (four items), inadequate rewards (three items), and organizational culture (four items). Each item was rated from 1 (not at all) to 4 (very much so), and a higher score indicated a higher level of JS. Regarding the tool's reliability, Cronbach's α values were 0.79 and 0.81 in Baik and Choi-Kwon's study [40] and this study, respectively.

#### 3.3.3. SOE

An instrument modified by Lee [41] and developed by Eisenberger et al. [42] was utilized. It comprised 21 items across three subfactors: perceived organizational support (nine items), perceived supervisor support (six items), and perceived coworker support (six items). Each item was rated on a 5-point Likert scale that ranged from 1 (not at all) to 5 (very much so). A higher score indicated a greater perception of a SOE. Cronbach's α values were 0.89, 0.92, and 0.89 for perceived organizational, supervisor, and coworker support in Lee's [41] study, respectively. In this study, Cronbach's α values were 0.93, 0.94, and 0.93, respectively.

#### 3.3.4. PO

The Psychological Ownership Questionnaire (POQ), developed by Avey et al. [28], was utilized. A Korean translation was available from https://www.mindgarden.com. It comprised 16 items: 12 for the promotive aspects, such as self-efficacy, responsibility, belongingness, and self-identity, and three for the defensive aspect of territoriality. This study utilized the tool modified by Cho et al. [43] for nurses, which comprised 12 items with three items each for self-efficacy, responsibility, belongingness, and self-identity. Responses were rated on a 6-point Likert scale that ranged from 1 (not at all) to 6 (very much so). A higher score signified a higher level of PO. The overall Cronbach's α for PO was 0.91, with subscale scores of 0.92 for self-efficacy, 0.89 for accountability, 0.81 for a sense of belonging, and 0.82 for self-identity [43]. In this study, the overall Cronbach's α was 0.92, with subscale scores of 0.92 for self-efficacy, 0.90 for accountability, 0.80 for a sense of belonging, and 0.80 for self-identity.

#### 3.3.5. CC

The CC instrument for nurses, developed by Kim et al. [44], was utilized. One item, “I devote a significant amount of time to reading nursing-related books,” was modified to “I spend a lot of time reading nursing-related literature (journals, textbooks, etc.)” to fit the nursing organizational context. Items were rated on a Likert 5-point scale that ranged from 1 (not at all) to 5 (very much so). Higher scores indicated a higher level of CC. Cronbach's α values were 0.83 and 0.86 in Kim et al.'s study [44] and this study.

#### 3.3.6. ProQoL

ProQoL was measured based on the Professional Quality of Life Scale by Stamm [5], modified and supplemented by Cho [45]. It comprised two subareas: CF (secondary traumatic stress and burnout) and CS. The instrument comprised 30 items, 20 on CF and 10 on CS. Items were rated on a 5-point Likert scale that ranged from 1 (not at all) to 5 (very much so). Higher scores represented higher levels of CF and CS, respectively. Cronbach's α values were 0.75 and 0.90 for CS and CF in Cho [45]'s study, respectively, and 0.83 (0.82 for burnout and 0.83 for secondary traumatic stress) and 0.94, respectively, in this study.

### 3.4. Data Collection and Ethical Consideration

This study was approved by the Institutional Review Board of the University the researcher was affiliated with and external institutions (IRB No: GIRB-A23-NY-0005, 2023-01-00-002). Data were collected between February 13 and 24, 2023. The researcher explained the study purpose, method, and procedures to the nursing department of the respective hospitals and obtained their consent. Instructions regarding precautions during questionnaire completion were provided, and cooperation was requested for distribution and collection. When the materials were distributed, the researchers explained the study's necessity and methods to the participants and included nurses who provided written consent. It was clarified that the data would be used solely for research purposes. Additionally, participants were informed that they could withdraw at any point without any disadvantage. Completed questionnaires were placed in opaque envelopes, sealed, and dropped into a collection box at the nurses' station to ensure anonymity. Subsequently, the researcher visited each department to collect the sealed questionnaires. After the questionnaires were collected, all participants were provided a small gift. Collected data were coded to protect personal information, and questionnaires were securely sealed and stored to safeguard participants' privacy. They will be retained for three years after the study's conclusion and destroyed by incineration.

### 3.5. Data Analysis

Data were analyzed using SPSS/WIN Version 23.0 and AMOS Version 21.0 (IBM Corp, Armonk, NY, USA). Descriptive statistics were used to assess participants' general characteristics and measurement variables. Differences in ProQoL between groups were analyzed using the independent *t*-test, one-way ANOVA, and Scheffe test for the post hoc test. Correlations between measurement variables were analyzed via Pearson's correlation coefficients. The normality of the sample was assessed via standardized skewness and kurtosis values. Multicollinearity among the latent variables was assessed via the variance inflation factor (VIF). Structural model validation was conducted via the maximum likelihood method. Furthermore, the convergent validity and discriminant validity of the latent variables were assessed via a confirmatory factor analysis. Several absolute fit indices were utilized to evaluate the fit of the hypothetical model, such as the chi-squared (*χ*^2^) test, normed *χ*^2^, the goodness-of-fit-index (GFI), and incremental fit indices, such as the Normed Fit Index (NFI) and Comparative Fit Index (CFI), Tucker–Lewis Index (TLI) as a relative fit index, and root mean square error of approximation (RMSEA) and root mean square residual (RMR).

Paths in the hypothesized model were assessed for significance via the standardized estimate, critical ratio, and *p* values. Explanatory power for the endogenous variables was assessed via squared multiple correlation (SMC). Bootstrapping was employed to assess the significance of the model's direct, indirect, and total effects. *p* < 0.05 was considered statistically significant.

## 4. Results

### 4.1. Participants' Sociodemographic Characteristics and Differences Between Groups in ProQoL

The number of hospital beds ranged mostly between 300 and 500 (73.3%), followed by those with 100–300 beds (26.7%). Experienced nurses' average age was 31.31 years. Additionally, 77.0% had a bachelor's degree (their highest attainment), and 66.9% were single. Regarding positions, 273 (92.2%) were staff nurses, followed by 23 (7.8%) charge nurses. Regarding work schedules, 257 (86.8%) had shift work, and 39 (13.2%) had non-shift work. The average clinical and departmental experience was 7.47 ± 5.63 and 3.57 ± 3.22 years, respectively. In addition, 84 individuals (28.4%) were satisfied with welfare benefits, while 63 (21.3%) were dissatisfied. Regarding job satisfaction, 156 individuals (52.7%) considered it as average, 86 (29.1%) were satisfied, and 54 (18.2%) were dissatisfied. The characteristics showing differences in CS were age (*F* = 3.03, *p*=0.030), education level (*F* = 3.85, *p*=0.022), total clinical experience (*F* = 4.16, *p*=0.007), welfare satisfaction (*F* = 18.01, *p* < 0.001), and job satisfaction (*F* = 48.29, *p* < 0.001). The characteristics showing differences in CF were total clinical experience (*F* = 4.39, *p*=0.005), welfare satisfaction (*F* = 6.94, *p*=0.001), and job satisfaction (*F* = 24.60, *p* < 0.001) (Table 1).

### 4.2. Descriptive Statistics of the Variables, Normality Tests, and Correlations

The JS score was 45.17 ± 8.94 (out of 100). SOE scored 3.43 ± 0.54 (out of 5 points), PO scored 3.90 ± 0.69 (out of 6 points), and CC scored 2.85 ± 0.72 points (out of 5 points). CC scored 2.85 ± 0.72 points (out of 5 points). For ProQoL, CF and CS scored 2.57 ± 0.51 and 3.46 ± 0.62 points, respectively. For JS, SOE, PO, CC, and ProQoL, the skewness ranged from −0.46 to 0.38 (within ±2), and kurtosis ranged from −0.78 to 1.41 (within ±7), which indicated that a normal distribution could be assumed. Analysis of the correlations among the variables revealed that CF exhibited the highest positive correlation with JS (*r* = 0.52, *p* < 0.001) and negative correlations with PO (*r* = −0.33, *p* < 0.001), CC (*r* = −0.39, *p* < 0.001), and CS (*r* = −0.44, *p* < 0.001). CS had the highest positive correlation with CC (*r* = 0.52, *p* < 0.001), followed by PO (*r* = 0.49, *p* < 0.001). Moreover, a SOE (*r* = 0.41, *p* < 0.001) also had a positive correlation (Table 2).

### 4.3. Validation of the Hypothesized Model

#### 4.3.1. Confirmatory Factor Analysis

A confirmatory factor analysis to verify the convergent validity of the latent variables was performed to ensure the standardized coefficients (β), construct reliability, and average variance extracted (AVE) were above 0.50, 0.70, and 0.50, respectively. All the standardized coefficients for the SOE, PO, and ProQoL were above 0.50. Additionally, construct reliability ranged from 0.88 to 0.90, which indicated a value above 0.80. The AVE ranged from 0.65 to 0.86, which indicated a value above 0.60. Thus, the convergent validity of the latent variables was confirmed. Additionally, discriminant validity results showed that the AVE values ranged from 0.65 to 0.76, which were greater than the squared correlation coefficients (0.11–0.47), indicating that the concepts were sufficiently distinct.

#### 4.3.2. Validation of the Hypothesized Model

Regarding the fit of the hypothesized model, a chi-square value of 130.81 (*p* < 0.001) did not satisfy the fit criterion. However, the normalized chi-square (chi-square/df) value of 2.84, absolute fit indices, SRMR (0.05), GFI (0.93), AGFI (0.89), and RMSEA (0.08), and incremental fit indices, NFI (0.92), TLI (0.92), and CFI (0.95), met the fit criteria (Figure 1). Therefore, the hypothesized model was the final model.

#### 4.3.3. Analysis of the Final Model

**Figure 3:**
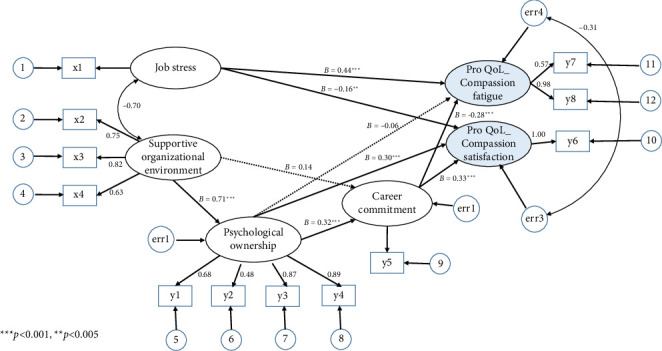
Final model of nurses' professional quality of life. Note: x1: job stress, x2: organizational support, x3: supervisor support, x4: peer support, y1: self-efficacy, y2: accountability, y3: belongingness, y4: self-identity, y5: career commitment, y6: compassion satisfaction, y7: secondary traumatic stress, and y8: burnout. Fit indices: *χ*^2^(*p*)=130.81 (*p* < 0.001); (*χ*^2^/df) = 2.84; SRMR (0.05); GFI (0.93); AGFI (0.89); RMSEA (0.08); NFI (0.92); TLI (0.92); CFI (0.95).

In the final model, seven of the nine paths were statistically significant, except from a SOE to CC and PO to CF (Figure 3 and Table 3). JS (*β* = 0.44, *p* < 0.001) and CC (*β* = −0.28, *p* < 0.001) significantly influenced CF. Although PO did not directly influence CF (*β* = −0.06, *p*=0.255), it had an indirect effect through CC (indirect effect = −0.09, *p*=0.008). Furthermore, the total effect was significant (total effect = −0.15, *p*=0.016). A SOE indirectly influenced ProQoL and CF and directly influenced PO and CC. The explanatory power of these variables for CF was 37% (SMC = 0.37).

JS (*β* = −0.16, *p*=0.003), PO (*β* = 0.30, *p* < 0.001), and CC (*β* = 0.33, *p* < 0.001) significantly impacted CS. SOE had a direct effect on PO (*β* = 0.71, *p* < 0.001), and PO had a direct effect on CC (*β* = 0.32, *p* < 0.001), which directly impacted CS. These observations demonstrated that SOE (indirect effect = 0.34, *p*=0.014) and PO (indirect effect = 0.11, *p*=0.004) indirectly influenced CS. The explanatory power of these variables was 39% (SMC = 0.39).

## 5. Discussion

The ProQoL model for career nurses, based on the expanded JD-R model [17], had an explanatory power of 37%–39%. In structural equation modeling, the SMC representing explanatory power did not have an absolute standard; however, approximately 40% was considered relatively good [46]. Therefore, this study's model could be considered acceptable for explaining and predicting nurses' ProQoL. It could serve as a meaningful theoretical framework to identify factors affecting nurses' quality of life in subsequent research.

JS and CC directly influenced CF, whereas JS, PO, and CC directly affected CS. JS was the most significant factor (*β* = 0.44) influencing CF. These findings supported previous research [14, 15], which suggested that job demands, including JS, significantly impacted CF and burnout. Therefore, devising strategies to decrease job-related stress, which will enhance ProQoL, is necessary to reduce CF among nurses. In this study, JS scored below the midpoint (average of 45.17 points). However, although not presented due to space constraints, the actual results show that among the subareas of JS, job demand scored the highest (70.02 points), followed by physical environment (58.61), inadequate rewards (46.42), organizational system (44.49), lack of job autonomy (44.43), workplace culture (35.89), job insecurity (32.06), and interpersonal conflict (29.09). A study [39] with three shift nurses, which included newly hired nurses at a general hospital in South Korea, used the same tool and reported an average JS score of 49.89 points, which was lower than that in this study. However, the study has similarly high scores for job demand (77.78), physical environment (69.25), and inadequate rewards (54.26 points). The study implied that JS related to high workloads and inadequate support and compensation due to a shortage of nursing staff was significant among nurses. Therefore, since JS lowered the ProQoL and negatively affected health, such as personal sleep and physiological functions [40], organizational support focused on mitigating JS is required. Additionally, to reduce job-related stress, it is necessary to improve the nursing work environment by reducing excessive workload, enhancing autonomy, and implementing stress reduction programs. In particular, it is essential to develop and implement targeted intervention programs that specifically address the key stressors identified in this study—namely, high job demands, inadequate rewards, and limited job autonomy.

In this study, PO did not directly impact CF in ProQoL; however, it had an indirect negative effect through CC. Additionally, it had a direct impact on CS and an indirect positive effect on CC. Research [47] on nurses from a hospital that provided integrated nursing and caregiving services, based on the JD-R model, reported that personal resources affected job burnout. Similarly, Xanthopoulou et al. [17] found that personal resources were significantly related to burnout, presenting a different view from this study. Nevertheless, the expanded JD-R model posited that personal and job resources could buffer the negative effects of burnout. In this study, PO had a direct impact on CS. It was also a mediating factor that enhanced CS through CC and mitigated CF, consistent with the JD-R model. Therefore, personal resources were an important factor in improving ProQoL.

In this study, PO had an average rating of 3.90, similar to the 3.97 found in a previous study [43]. This score indicated that nurses' sense of PO toward the organization they belonged to was between “generally not the case” and “generally the case.” Since PO is closely related to the nursing work environment and organizational atmosphere [48], strategies and influencing factors should be explored to enable nurses to develop a sense of PO. Also, for nursing management, fostering a sense of belonging and ownership among nurses by creating opportunities for participation in decision-making processes, offering continuous feedback, and recognizing their contributions are proposed. Building a supportive organizational culture can further strengthen PO.

This study identified CC as a major factor influencing CF and CS. Moreover, CC played a mediating role in a SOE and CS, as well as between PO, CS, and fatigue. Although direct comparisons were difficult owing to the rarity of prior research on the effect of CC on ProQoL, studies reported a negative relationship between CC and intent to leave [44] and burnout [24] and a positive relationship with CS [25]. Although the study was on childcare teachers [38], stress was related to reduced CC (*β* = −0.26) and increased burnout (*β* = 0.48). Additionally, increased CC led to decreased burnout (*β* = −0.36), which thereby partially supported the results of this study. In this study, participants' CC was 2.85 out of 5 points, which was considered below “average.” Compared to other studies that used the same instrument, CC among nurses in general hospitals with more than 300 beds, three smaller hospitals with less than 300 beds, and a university hospital was 3.05 [49], 3.17 [50], and 2.59, respectively [44]. Therefore, there were differences in nurses' CC based on the type of hospital. In particular, nurses in smaller hospitals had relatively higher CC. However, further research was necessary to determine the causes and the severity of patient conditions and the fact that work burdens were relatively low in general hospitals. Some nurses were transferred from high-level hospitals, which could contribute to their higher attachment to and involvement in their careers. Therefore, nursing organizations should develop programs and support systems that encourage CC and enable nurses to stay at a job for an extended period.

Furthermore, hospital-level improvements are also essential. Meticulous attention is required to ensure that nurses can commit to their careers. CC, expressed as pride in one's profession and a sense of unity, is an essential key factor determining employees' happiness or quality of work life. Maslow reported that pride and commitment to one's profession were necessary conditions for self-actualization, the highest need humans aspire to fulfill [24].

CC was influenced by emotional dispositions, such as self-esteem, and organizational environmental factors, such as social support, namely, support from supervisors and human resource management support [24, 25]. Additionally, an open organizational atmosphere that embraced diversity and offered many growth opportunities increased CC among members [49]. Therefore, to retain competent career nurses and encourage long-term employment, offering opportunities for participation in hospital issues and decision making, providing continuous feedback, and fostering an environment that recognizes and cultivates nurses are necessary. Moreover, nurses' CC should not be only a requirement at the beginning; it should be continuously developed. Nursing organizations should assist in maintaining highly committed nurses so they do not leave their positions and develop strategies to enhance nurses' CC. Physical support should also be available for nurses to continue their career development.

In this study, a SOE indirectly influenced ProQoL through PO and CC. These results were partially similar to those of Cho's study [51], which targeted nurses who worked in tertiary general and general hospitals and suggested that social support, a job resource, affected CS. Moreover, as presented in the expanded JD-R model [17], job resources affected burnout indirectly through personal resources without a direct path to burnout; thus, these results validated the expanded JD-R model. Job resources, such as a SOE, indirectly influenced ProQoL or directly through personal resources, such as PO and CC. Therefore, to enhance nurses' ProQoL, a SOE that fosters PO and CC should be prioritized. Participants' perceptions of a SOE were at 3.43 on a 5-point scale above the midpoint. Regarding the subdomains, the perception of peer support was the highest (3.84), followed by supervisor (3.55) and organizational support (3.09). This was consistent with a study [41] that targeted nurses at a university hospital, which also found that organizational support perception was the lowest, which indicated a greater need for organizational support. Regular assessment of organizational support perceptions can guide targeted interventions to improve nurses' ProQoL.

Participants' level of ProQoL was 2.57 for CF and 3.46 for CS. CS was lower among those aged 25–36 years, with total clinical experience ranging from > 3–5 years to 5–10 years. CF was higher among those with total clinical experience of 5–10 years. Previous studies have reported varying results regarding the association between general characteristics and ProQoL. One study reported that older nurses experienced higher CS [52], while another found that younger nurses reported higher levels of CF [53]. These findings differ from the results of the present study. In this study, CF was significantly higher when welfare benefits and job satisfaction were low. Additionally, nurses with 5–10 years of experience showed lower CS and higher CF. Hence, efforts to improve welfare and treatment to enable professionals with these career lengths to feel job satisfaction and remain employed over the long term are necessary. This study did not compare cultural differences in South Korea, but South Korean nursing organizations are generally relationship-oriented, which is positively correlated with CS and negatively correlated with burnout [53]. Therefore, future studies are needed to compare ProQoL based on cultural and value differences.

The study underscores the need for holistic approaches to improving ProQoL by addressing both environmental and personal resources, encouraging long-term CC, and reducing JS. However, this study was limited to nurses in regional general hospitals with a small sample size; the results should be generalized with caution. Additionally, concepts such as a supportive environment, PO, CS, and fatigue cannot exclude the possibility of social desirability bias among participants.

## 6. Implications for Nursing Management

This study developed a ProQoL model for hospital career nurses based on the expanded JR-D model [17]. The model provided an appropriate theoretical foundation to explain nurses' ProQoL. The study identified JS, a SOE, PO, and CC as factors influencing career nurses' ProQoL. Therefore, nursing organizations should support JS reduction for career nurses through improvements in the working environment, provision of appropriate compensation, and continuous feedback. Creating an environment that acknowledges and nurtures nurses will allow them to participate in problem solving and decision making regarding hospital issues, thus enhancing PO within the organization. Additionally, there is a need to create and implement CC development programs along with emotional and physical support to enable CC.

## 7. Conclusion

This study found that the expanded JR-D model for nurses was suitable to explain and predict ProQoL. Factors influencing nurses' ProQoL included JS, a SOE, and CC. Therefore, the established model should be applied to developing nursing intervention programs to improve nurses' ProQoL. Follow-up studies are required to confirm ProQoL by expanding the scale and region of hospitals. Repeated research is required to determine the effects of various job demands, job resources, and personal resource variables on ProQoL beyond the variables validated in this study. Follow-up studies should aim to develop programs at the organizational level to enhance PO and CC, as well as verify the intervention effects to increase empathic satisfaction and decrease empathic fatigue. The results of these studies can improve nurses' ProQoL. Future qualitative research should aim to understand the in-depth causes and phenomena of CC among nurses in general hospitals.

## Figures and Tables

**Table 1:** Participants' sociodemographic characteristics and differences in ProQoL (*N* = 296).

Characteristics	Categories	*n*	%	Professional quality of life					
				Compassion fatigue			Compassion satisfaction		
				*M* ± SD	*t*/*F*	*p* (Scheffe)	*M* ± SD	*t*/*F*	*p* (Scheffe)
Gender	Male	19	6.4	2.38 ± 0.57	−1.72	0.086	3.74 ± 0.89	1.46	0.162
	Female	277	93.6	2.59 ± 0.51			3.44 ± 0.59		

Age (yr) (*M* ± SD: 31.31 ± 6.02)	≤ 25^a^	45	15.2	2.48 ± 0.47	1.10	0.349	3.52 ± 0.56	3.03	0.030
	26–30^b^	119	40.2	2.60 ± 0.51			3.42 ± 0.64		*d* > *c*
	31–35^c^	69	23.3	2.63 ± 0.46			3.34 ± 0.53		
	≥ 36^d^	63	21.3	2.52 ± 0.59			3.64 ± 0.66		

Educational level	3-year college^a^	45	15.2	2.52 ± 0.49	2.91	0.056	3.47 ± 0.59	3.85	0.022
	4-year college^b^	228	77.0	2.61 ± 0.49			3.42 ± 0.62		*c* > *a*, *b*
	≥ graduate school^c^	23	7.8	2.35 ± 0.67			3.80 ± 0.60		

Marital status	Married	98	33.1	2.51 ± 0.54	−1.37	0.170	3.59 ± 0.59	2.63	0.009
	Single	198	66.9	2.60 ± 0.49			3.39 ± 0.62		

Number of hospital beds	100∼< 300	79	26.7	2.56 ± 0.53	−0.27	0.791	3.43 ± 0.52	−0.61	0.542
	300∼< 500	217	73.3	2.58 ± 0.51			3.47 ± 0.65		

Position	Staff nurse	273	92.2	2.59 ± 0.51	1.57	0.118	3.44 ± 0.61	−1.95	0.052
	Charge nurse	23	7.8	2.41 ± 0.54			3.70 ± 0.68		

Work pattern	3 shifts	257	86.8	2.57 ± 0.49	−0.34	0.734	3.44 ± 0.62	−1.17	0.243
	Day fixed shift	39	13.2	2.60 ± 0.63			3.57 ± 0.61		

Work unit	General ward	151	51.0	2.59 ± 0.49	1.48	0.220	3.45 ± 0.62	1.69	0.169
	Nursing care integrated ward	52	17.6	2.66 ± 0.47			3.34 ± 0.58		
	ICU/ER/OR/DR	79	26.7	2.51 ± 0.52			3.52 ± 0.59		
	Others	14	4.7	2.40 ± 0.78			3.71 ± 0.75		

Total clinical experience (yr) (*M* ± SD: 7.47 ± 5.63)	1 < 3^a^	73	31.9	2.46 ± 0.47	4.39	0.005	3.62 ± 0.63	4.16	0.007
	3∼5^b^	48	21.0	2.53 ± 0.54		*c* > *a*	3.34 ± 0.62		*a* > *d*
	5∼10^c^	91	39.7	2.72 ± 0.43			3.33 ± 0.53		
	≥ 10^d^	17	7.4	2.69 ± 0.53			3.25 ± 0.68		

Current unit experience (yr) (*M* ± SD: 3.57 ± 3.22)	> 1	51	17.2	2.55 ± 0.48	0.50	0.681	3.51 ± 0.65	0.89	0.447
	1∼2	76	25.7	2.59 ± 0.44			3.53 ± 0.57		
	2∼5	86	29.1	2.53 ± 0.61			3.38 ± 0.71		
	≥ 5	83	28.0	2.62 ± 0.48			3.45 ± 0.53		

Satisfaction with the hospital's welfare	Unsatisfactory^a^	63	21.3	2.73 ± 0.47	6.94	0.001	3.19 ± 0.52	18.01	< 0.001
	Usually^b^	149	50.3	2.59 ± 0.48		*a* > *c*	3.41 ± 0.62		*c* > *a*, *b*
	Satisfaction^c^	84	28.4	2.42 ± 0.56			3.76 ± 0.57		

Job satisfaction	Unsatisfactory^a^	54	18.2	2.87 ± 0.48	24.60	< 0.001	3.05 ± 0.62	48.29	< 0.001
	Usually^b^	156	52.7	2.61 ± 0.49		*a* > *b* > *c*	3.36 ± 0.49		*c* > *b* > *a*
	Satisfaction^c^	86	29.1	2.31 ± 0.44			3.90 ± 0.55		

Abbreviations: DR = delivery room; ER = emergency room; ICU = intensive care unit; OR = operating room.

^a, b, c, d^Values with different superscript letters are significantly different (*p* < 0.05) by Scheffe's post hoc test.

**Table 2:** Descriptive statistics of the variables, normality tests, and correlations.

Variables		*M* ± SD	Skewness	Kurtosis	Job stress	Supportive organizational environment	Psychological ownership	Career commitment	ProQoL	
									Compassion satisfaction	Compassion fatigue
					*r* (*p*)					
Job stress		45.17 ± 8.94	0.10	1.08	1					

Supportive Organizational Environment		3.43 ± 0.54	0.10	0.11	−0.60⁣^∗^	1				

Psychological Ownership		3.90 ± 0.69	−0.38	0.87	−0.47⁣^∗^	0.55⁣^∗^	1			

Career commitment		2.85 ± 0.72	0.04	0.12	−0.37⁣^∗^	0.31⁣^∗^	0.37⁣^∗^	1		

ProQoL	Compassion satisfaction	3.46 ± 0.62	0.06	0.12	−0.44⁣^∗^	0.41⁣^∗^	0.49⁣^∗^	0.52⁣^∗^	1	
	Compassion fatigue	2.57 ± 0.51	−0.09	0.28	0.52⁣^∗^	−0.25⁣^∗^	−0.33⁣^∗^	−0.39⁣^∗^	−0.44⁣^∗^	1

⁣^∗^*p* < 0.001.

**Table 3:** Parameter estimates for hypothetical structural model and standardized direct, indirect, and total effects.

Endogenous variables		Exogenous variables	*B*	S.E	*β*	CR	*p*	SMC	Direct effect (*p*)	Indirect effect (*p*)	Total effect (*p*)
Psychological ownership		Supportive organizational environment	1.31	0.14	0.71	9.15	< 0.001	0.50	0.71 (< 0.001)	—	0.71 (< 0.001)

Career commitment		Supportive organizational environment	0.26	0.17	0.14	1.50	0.134		0.14 (0.134)	0.23^a^ (0.003)	0.37 (0.021)
		Psychological ownership	0.32	0.09	0.32	3.54	< 0.001		0.32 (< 0.001)	—	0.32 (< 0.001)

ProQoL	Compassion fatigue	Job stress	0.01	0.00	0.44	4.81	< 0.001	0.37	0.44 (< 0.001)	—	0.44 (< 0.001)
		Supportive organizational environment	—	—	—	—	—		—	−0.15^b^ (0.009)	−0.15 (0.009)
		Psychological ownership	−0.02	0.02	−0.06	−1.14	0.255		−0.06 (0.255)	−0.09 (0.008)	−0.15 (0.016)
		Career commitment	−0.11	0.03	−0.28	−4.19	< 0.001		−0.28 (< 0.001)	—	−0.28 (< 0.001)
	Compassion satisfaction	Job stress	−0.01	0.00	−0.16	−2.95	0.003	0.39	−0.16 (0.003)	—	−0.16 (0.003)
		Supportive organizational environment	—	—	—	—	—		—	0.34^c^ (0.014)	0.34 (0.014)
		Psychological ownership	0.26	0.05	0.30	4.89	< 0.001		0.30 (< 0.001)	0.11 (0.004)	0.41 (0.012)
		Career commitment	0.29	0.04	0.33	6.50	< 0.001		0.33 (< 0.001)	—	0.33 (< 0.001)

*Note: B* = unstandardized coefficient; *β* = standardized estimate; ProQoL = professional quality of life. Indirect effect: a = supportive organizational environment ⟶ psychological ownership ⟶ career commitment; b = supportive organizational environment ⟶ psychological ownership ⟶ career commitment ⟶ compassion fatigue; c = supportive organizational environment ⟶ psychological ownership ⟶ career commitment ⟶ compassion satisfaction.

Abbreviations: CR = construct reliability; S.E = standard error; SMC = squared multiple correlation.

## Data Availability

The data used to support the findings of this study have not been made available due to confidentiality issues.

## Supporting Information

This study includes the following Supporting Information. Supporting File 1 contains the STROBE checklist for the reporting of observational studies.
